# A four-level model of political polarization over science: Evidence from 10 European countries

**DOI:** 10.1177/09636625241306352

**Published:** 2025-01-18

**Authors:** Roderik Rekker

**Affiliations:** Radboud University, The Netherlands; University of Gothenburg, Sweden; Leibniz University Hannover, Germany

**Keywords:** ideology, polarization, populism, science skepticism, trust

## Abstract

Citizens’ trust in science increasingly depends on their political leaning. Structural equation models on survey data from 10 European countries (*N* = 5306) demonstrate that this *science polarization* can be captured by a model with four levels of generalization. Voters of populist parties distrust the *system and elite* in general, which indirectly fuels a broad science skepticism. At another level, right-wingers have less trust in *science as a whole* than left-wingers. After accounting for this general skepticism, left-wingers and right-wingers are, however, similarly prone to contest ideology-incongruent *research fields* and *specific claims*. These findings have three implications. First, research on science skepticism should carefully consider all four levels and their interplay. Second, the science polarization between populist and non-populist voters has fundamentally different origins than the effect of left–right ideology. Third, a four-level model can expose ideological symmetries in science rejection that have previously remained largely undetected in observational studies.

## 1. Introduction

**Figure 1.**
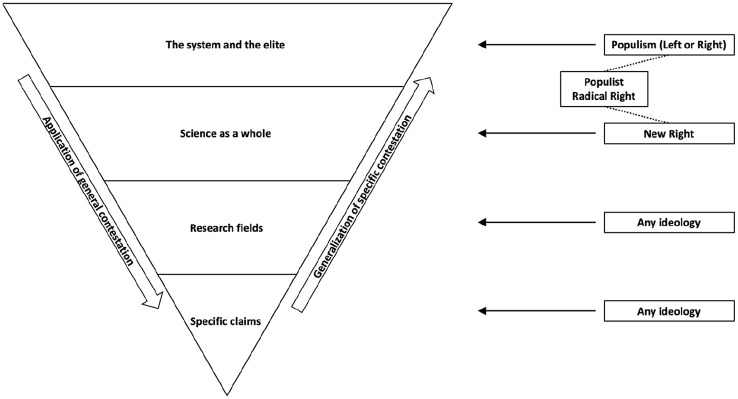
Political polarization over science as a four-level model. Source: Adopted from Rekker (2021).

Citizens’ trust in science, as well as their factual beliefs more generally, are increasingly connected to their political ideology and party preference (Gauchat, 2012; Oreskes and Conway, 2022; Rekker, 2021, 2022). On a variety of issues such as COVID-19 and climate change, citizens’ acceptance of scientific knowledge depends on where they stand on the political spectrum (Dunlap et al., 2016; Kerr et al., 2021). Although the association between political leaning and science rejection is well established, this article argues that the existing literature has failed to provide a definitive answer to the crucial question where, exactly, this *science polarization* originates from. Rekker (2021) argued that science rejection can originate from four distinct levels of generalization as depicted in Figure 1. Citizens can reject *specific claims* such as that global warming is human-made (Level 1), but also entire *research fields* such as environmental science (Level 2). Moreover, people can distrust *science as a whole* (Level 3) or, even more generally, the *system and elite* (Level 4). However, the empirical evidence for this model is currently incomplete because previous studies have examined only one or two of these levels at once.

This study fills this void by using structural equation modeling to investigate science polarization on all four levels simultaneously with new survey items that were included in the “CRONOS-2 panel.” This online panel was fielded among 5306 respondents from 10 European countries (Austria, Belgium, Czechia, Finland, France, Italy, Portugal, Slovenia, Sweden, and the United Kingdom) who previously participated in the European Social Survey (ESS). Respondents were inquired about their trust in six scientific claims (i.e. anthropogenic global warming, the social origins of gender roles, the safety of genetically modified foods, the relationship between tax rates and tax revenue, the application of antibiotics, and the expanse of the universe), the six research fields that made these claims, (i.e. environmental science, sociology, agricultural science, economics, medical science, and physics), three facets of science as a whole (i.e. universities, scientists, and the scientific method), and seven elite institutions (i.e. the national parliament, the legal system, the police, politicians, political parties, the European Parliament, and the United Nations). By simultaneously examining how these four levels of trust relate to citizens’ left–right ideology and support for populist parties, this study is uniquely positioned to answer questions such as whether right-wingers contest global warming above and beyond their rejection of environmental science as a field or whether populist voters distrust science more than should be expected based on their overall distrust in elite institutions.

The theoretical contribution of this study is twofold. First, this examination can—assuming a direction of causality in cross-sectional data—trace back science polarization to the level of contestation from which it originates. A better understanding of these origins can inform strategies to counteract science polarization. For example, if populist and non-populist voters are divided mainly over their general trust in the political system—and reject climate science only as a byproduct—the solution may be found mostly in bridging the gap between elites and ordinary citizens more generally, rather than in specific science communication strategies. Second, this study’s four-level model contributes to the ongoing debate about the *ideological symmetry* of science rejection. Some scholars have argued that right-wingers are universally more likely to reject science than people on the left (e.g. Kruglanski, 2004; Mooney, 2012), whereas others have claimed that both sides of the political spectrum are equally predisposed to reject science when it contradicts their ideological worldview (e.g. McCright et al., 2013; Nisbet et al., 2015). Drawing from Rekker’s (2021) theoretical model (see Figure 1), this study aims to reconcile both positions by proposing that both leftist and rightist citizens tend to distrust ideology-incongruent scientific claims and fields but that only right-wingers and populist voters are predisposed to reject science as a whole or the entire system and elite. Finally, this study makes an empirical contribution by comparing science polarization in 10 European countries, whereas previous studies have focused mostly (albeit not exclusively, e.g. Czarnek et al., 2021) on the United States.

## 2. Theory and hypotheses

### Level 1: Specific claims

A first level of political polarization involves specific scientific claims. Survey research has, for instance, established unequivocally that conservatives are more likely than liberals to question anthropogenic climate change (McCright and Dunlap, 2011). There is, however, no consensus whether there is *ideological symmetry* in science rejection or, in other words, whether liberals are equally predisposed to reject identity-incongruent science. On one side of this debate, the *intrinsic thesis* posits that conservatives are characterized by distinct personality traits that make them more likely than liberals to disregard information that challenges their worldview (Jost et al., 2003; Kruglanski, 2004). This account is supported by studies that revealed cognitive differences between liberals and conservatives in which the latter were more likely to avoid dissonant information (e.g. Nam et al., 2013; Shook and Fazio, 2009). The intrinsic thesis has, however, been critiqued based on the validity and relevance of the reasoning measures that conservatives score lower on, as well as on the internal validity of its experiments (Kahan, 2012, 2016). More recent studies have, therefore, relied on more stringent measures and designs. Challenging the intrinsic thesis, a meta-analysis of such studies revealed that liberals and conservatives are equally biased in their cognition of counterattitudinal information (Ditto et al., 2019). As an alternative, the *contextual thesis* proposes that the association between political leaning and science rejection is driven by an interplay between citizens’ political identity and the politicization of science by elites (Newman et al., 2018; Nisbet et al., 2015). According to this perspective, both liberals and conservatives are predisposed to reject identity-incongruent information due to a psychological need to protect beliefs that maintain their status in an affinity group (Cohen, 2003). This idea is supported by experiments in which the identity congruence of stimulus material was manipulated, which altered information processing among both liberals and conservatives (Kahan, 2016; Washburn and Skitka, 2018).

**Figure 2.**
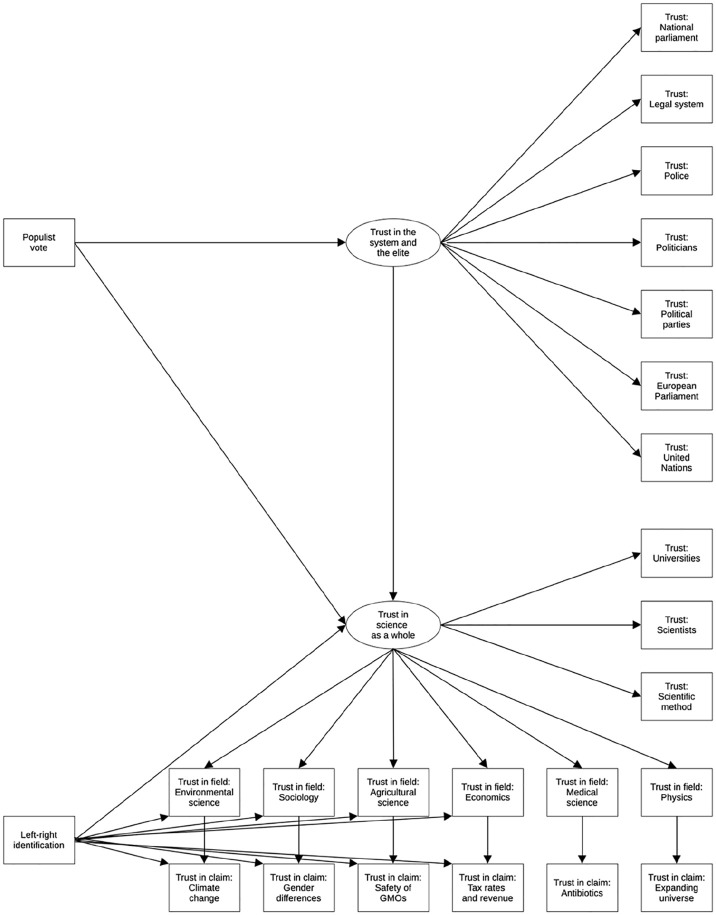
The hypothesized four-level model of science polarization.

Although the debate about the ideological symmetry of motivated reasoning is still ongoing (Jost, 2017), there is thus considerable evidence that liberals and conservatives are equally biased against counterattitudinal information. As such, one might expect that both sides of the political spectrum are also equally predisposed to distrust scientific claims that challenge their ideology. However, this is not the picture that emerges from surveys on *domain-specific science skepticism* (e.g. Rutjens et al., 2022) in which conservatives quite consistently express more skepticism than liberals. Liberals not only report more trust in claims that support their ideology, but also in more ideologically neutral claims or even claims that seemingly contradict their liberal worldview (Hunt and Wald, 2020; Pechar et al., 2018; Stein et al., 2021). A possible explanation for this paradox is that previous studies did not fully distinguish between the aforementioned levels of science polarization. That is, liberals are more likely to trust science as a whole than conservatives (Gauchat, 2012), which may in turn make them more likely to trust any scientific claim regardless of its content. This *positive indirect effect* might undo or reverse any *negative direct effect* of liberal ideology on trust in liberal-dissonant claims. To distinguish both processes, the effect of ideology on domain-specific science skepticism should be estimated while fully controlling for all three other levels of science polarization. To this end, the current examination will estimate a four-level model (see Figure 2) that can incorporate the effect of ideology on trust in science on each level while accounting for the others. In other words, this study can examine whether liberals express less trust in liberal-dissonant scientific claims than should be expected based on their higher overall trust in science.

The CRONOS-2 panel asked participants about their trust in two conservative-dissonant claims, two liberal-dissonant claims, and two neutral claims. The first conservative-dissonant claim is the notion that climate change is human-made, which may threaten conservatives’ ideological identity due to its implications for regulatory policies (Kahan, 2016). In addition, conservatives may take cues from conservative politicians and media that contest science on this issue (Rekker, 2021). A second conservative dissonant claim is the sociological idea that behavioral gender differences are the result of upbringing and society more than biology. The first liberal-dissonant claim is the notion that genetically modified organisms (GMOs) can improve the productivity of farming without posing health risks for consumers. This idea seems to challenge liberals’ ideology, which generally prioritizes public health and environmental protection over economic growth (Reisner, 2001). Nonetheless, previous studies—which did not fully control for more general levels of trust—revealed that liberals are *more* likely to trust science on the safety of GM foods than conservatives (Hunt and Wald, 2020; Pechar et al., 2018) and that they also have more accurate beliefs on this issue (McFadden, 2016; Pennycook et al., 2023; Rutjens et al., 2018). A second liberal-dissonant claim is the economic idea that increasing taxes on the highest incomes can reduce government revenue because people will decide to work less. This so-called “Laffer Curve” has frequently been challenged by left-wing parties (Berman and Milanes-Reyes, 2013). Economists generally agree that raising tax rates *can* decrease government revenue *after a certain point* (Jacobs, 2013), although most economists dispute Laffer’s claim that raising taxes *will* decrease revenue *from their current level* in the United States (Clark Center, 2012). The two politically neutral claims in this study are the notion that antibiotics are only effective against bacteria and the idea that the universe is expanding. The first hypothesis is postulated as follows:

H1: After accounting for more generalized levels of trust, right-wingers are more likely to reject conservative-dissonant claims (H1a), whereas left-wingers more often distrust liberal-dissonant claims (H1b), and both groups trust neutral claims equally (H1c).

### Level 2: Research fields

Liberals and conservatives may also disagree on the relevance and legitimacy of different scientific endeavors, which is why a second level of political contestation involves research fields. Most notably, a study by McCright et al. (2013) demonstrated that conservatives express less trust than liberals in *impact science* fields that examine the consequences of economic production for the environment and public health. Liberals, conversely, reported less trust in *production science* fields that develop innovations for economic production. This pattern supports the *anti-reflexivity thesis*, which proposes that conservatives’ opposition to science is motivated by an ideological agenda to defend the industrial capitalist order against scientific claims that the current economic system threatens the environment and public health (McCright and Dunlap, 2010). Following McCright et al. (2013), the current study examines the effect of political ideology on citizens’ trust in the impact science field of environmental science and the production science field of agricultural science. In the context of a four-level model of science polarization (see Figure 1), this effect is estimated while statistically controlling for trust in science as a whole, which may affect citizens’ trust in any research field.

Whereas McCright et al. (2013) focused on the natural sciences and technology, the present examination adds two social science fields, namely economics and sociology. The field of economics may be more controversial to liberals because it could be perceived as a social equivalent of production science. The question how nations and businesses can achieve economic growth has historically been a core pillar of economic inquiry, even though the field is much broader nowadays (Blaug, 1997). In the eyes of the public, economics may also be associated with some controversial right-wing policies—such as the downsizing of the public sector or the austerity measures during the financial crisis—that were implemented under the auspices of economists. Indeed, a survey revealed that economically leftist Norwegians have less trust in economics than right-wingers (Schrøder, 2023). The field of sociology, on the other hand, could be contested by conservatives more than by liberals due to its research on issues such as the reproduction of inequality or the social origins of crime and gender roles. More generally, the way sociologists think about the world may be at odds with the individualism of certain right-wing ideologies, as illustrated by Margaret Thatcher’s famous quote “There is no such thing as society. There are individual men and women, and there are families.” Indeed, previous research has shown that people tend to perceive sociologists as being left-leaning and economists as right-leaning, and that such perceived political orientations are associated with trust in research fields (Altenmüller et al., 2024; Clark et al., 2023).

Finally, this study includes two comparatively nonpoliticized research fields, namely medical science and physics. There is no apparent reason why political ideology would affect citizens’ trust in these fields above and beyond the aforementioned indirect effect via more generalized levels of trust in institutions and science. Nonetheless, a previous study yielded mixed findings in this regard by revealing that trust in nonpoliticized fields is associated with pro-environment attitudes, but not economic ideology, even after controlling for political trust (Schrøder, 2023). In sum, the present study examines the following hypothesis:

H2: After accounting for more generalized levels of trust, right-wingers are more likely to reject environmental science and sociology (H2a), while left-wingers more often distrust agricultural science and economics (H2b), and both groups trust medical science and physics equally (H2c).

### Level 3: Science as a whole

A third level of polarization involves science as a whole. At this level, surveys have consistently established that—at least in North America and Western Europe—people on the political right express less trust than left-wingers, even though this was not yet the case until a few years or decades ago (Cologna et al., 2024; Gauchat, 2012; McCright et al., 2013). There are several explanations for this clear ideological asymmetry in the rejection of science as a whole. Focusing specifically on the American case, Mooney (2005) argued that science polarization has been driven by the emergence of the “New Right” which, as an electoral alliance between the Religious Right and transnational corporations, may have had a wide array of political and ideological motivations to oppose science. Rekker (2021) furthermore reasoned that the conservative ideology has historically been more critical about the central role of science in modern societies, for example by questioning the idea that science should have a monopoly on truth claims or that it should be used as a driver of rapid and rational societal change. Moreover, Nisbet et al. (2015) proposed that conservatives may have developed a more general distrust of science because conservative-dissonant issues—such as climate change and human evolution—are currently much more salient than liberal-dissonant issues.

Another ideology that has been associated with the rejection of science as a whole is populism, which pits ordinary citizens against a corrupt elite (Mudde, 2007). In recent years, many right-wing populist parties made climate skepticism into a core pillar of their ideology, albeit in different ways and to varying degrees (Fraune and Knodt, 2018; Gardiner, 2019; Lockwood, 2018; Schwörer and Fernández-García, 2023). Lockwood (2018) theorized that right-wing populists see climate change mitigation as a liberal agenda that is pursued against the national interest by a cosmopolitan elite and, moreover, that the complexity of science may challenge their desire for simple political solutions based on “the will of the people.” Mede and Schäfer (2020) related political populism to “science-related populism,” which they conceptualized as the idea that ordinary people have a virtuous “common sense” and should therefore take the sovereignty over science and truth away from an unvirtuous academic elite. In some (but not all) countries, this anti-scientific stance intensified during the COVID-19 pandemic, when some populist parties rejected the scientific basis for containment measures by, for example, questioning the severity of the virus or the safety of vaccines (Crulli, 2021; Ringe and Rennó, 2023). Unsurprisingly, the voters of populist parties consistently express less trust in science as a whole than supporters of non-populist parties (Rekker, 2023). The third hypothesis is therefore postulated as follows:

H3: After accounting for more generalized levels of trust, right-wingers (H3a) and populist voters (H3b) are more likely to distrust science as a whole.

### Level 4: The system and elite

The fourth level of polarization involves the entire system and elite. For some people, science skepticism may be part of a broader discontent with politics, the elite, and its institutions. Surveys indicate that such distrust is associated with economically left-wing (Van der Meer and Hakhverdian, 2017) and culturally right-wing attitudes (Citrin et al., 2014). The general left–right dimension, however, is not strongly or consistently connected to institutional distrust (Van der Brug and Van Praag, 2007). Instead, citizens’ trust in elites and institutions is associated much more with whether or not they support populist parties and ideas (Rekker, 2023). This connection is unsurprising, given that the populist ideology claims that politics is about a fundamental divide between a corrupt elite and a homogeneous people (Mudde, 2007). Experiments and panel studies furthermore indicate that distrust in public institutions can be both a cause and a consequence of citizens’ decision to support a populist party (Rekker, 2023; Rooduijn et al., 2016, 2017). Some distrustful voters choose a populist party because it voices their discontent, but others initially support a populist party for other reasons and only later adopt their anti-elite rhetoric. The present study therefore examines the following hypothesis:

H4: Populist voters are more likely to distrust the system and elite.

### A four-level model of science polarization

The previous sections discussed how citizens’ political leaning can affect their trust in science on four levels of generalization. Crucially, these levels are all connected because trust may easily travel from one level to another. Rekker (2021) introduced the term *application of general contestation* for situations in which people use more general levels of trust as a heuristic to form more specific attitudes. Indeed, several studies established that more general levels of science skepticism play an important role in the acceptance of specific scientific claims (Brossard and Nisbet, 2007; Hmielowski et al., 2014). Following this logic, this study hypothesizes a causal chain in which citizens’ general level of institutional trust affects their trust in science as a whole, which in turn influences their trust in research fields, which in turn affects their trust in specific claims.

Because of this hypothesized causal chain, tracing back the origins of science polarization requires that all four levels of contestation are modeled simultaneously. When a populist voter expresses distrust in the notion of anthropogenic global warming, for instance, this may indicate a rejection of this specific claim (Level 1), but also skepticism about the field that made this claim (Level 2), distrust in science a whole (Level 3), or an even broader discontent with elite institutions (Level 4). Because no previous study has yet examined all four levels simultaneously, the present study is—within the limits of observational research—uniquely positioned to fully trace back science polarization to its origins. Previous studies, which examined only two levels at once, indeed provide some evidence for the partial mediation of the effect of political leaning on science rejection by more generalized levels of distrust. For example, some studies have established that the association between populist voting and science skepticism (Level 3) is partly, but not entirely, mediated by distrust in political institutions (Huber et al., 2022; Rekker, 2023). In a similar vein, another study (Pechar et al., 2018) found that the political preference of American (but not German) citizens predicts their trust in a variety of scientific claims (Level 1) partly via their trust in the government (Level 4). Other studies demonstrated that political divisions over specific scientific claims (Level 1) are partly accounted for by general science skepticism (Level 3; e.g. Kossowska et al., 2021) and trust in research fields (Level 2, e.g. Krange et al., 2021). Drawing from these previous findings, this study tests a model—which combines hypotheses 1 through 4—about the level from which different manifestations of science polarization originate (see Figure 2). For example, the polarization over climate change between leftist and rightist citizens is expected to originate from levels 1 through 3, whereas the division between populist and non-populist voters on this same issue is expected to originate from levels 3 and 4. This hypothesis can be postulated as follows:

H5: The hypothesized four-level model provides an accurate and parsimonious representation of how a variety of trust indicators relate to each other and to political leaning.

This hypothesis will be tested by using structural equation modeling to fit the complete hypothesized model in Figure 2 on the data. Hypothesis 5 is confirmed if this model fits the data without the need to add unexpected parameters. As such, this study provides the first complete empirical test of Rekker’s (2021) four-level model of science polarization.

## 3. Data and measures

This study uses original data (*N* = 5306) from the “In science we trust” item battery that was administered between June 2022 and February 2023 as part of the fifth wave of the CRONOS-2 panel. This online panel was fielded among respondents from 12 countries who had previously been part of the random probability sample of European Social Survey (ESS) Round 10. Depending on the country, this edition of the ESS was fielded between September 2020 and September 2022 using face-to-face interviews or self-completion questionnaires. Because wave 5 of the CRONOS-2 panel was not fielded in Hungary and no party-level data on populism was available for Iceland, 10 of the 12 countries could be included in the analyses: Austria, Belgium, Czechia, Finland, France, Italy, Portugal, Slovenia, Sweden, and the United Kingdom. All available data points are included in the analysis using “full information maximum likelihood.”

The first block of the “In science we trust” item battery was introduced with the sentence: “Let’s start with some questions about how much trust you have in different institutions or activities.” Respondents then indicated their trust in universities, scientists, and the scientific method (see Achterberg et al., 2017). The second block was introduced with the words “The next questions are about how much trust you have in specific scientific disciplines.” This introduction was followed by the question “How much trust do you have in this scientific discipline: Sociology” which was repeated for economics, physics, medical science, agricultural science, and environmental science. The third block was introduced with the sentence: “The next questions are about statements which scientists from different disciplines have made” followed by the question: “How much trust do you have in this statement made by scientists: Differences in behavior between men and women are not fixed at birth but are mainly caused by upbringing of parents and society.” This question was repeated for the statements: “Increasing taxes on the rich will reduce government revenue because rich people will work less,” “The universe expands at an increasing rate,” “Antibiotics do not work against viruses because they only kill bacteria,” “The Earth’s climate is changing as a result of greenhouse gas emissions caused by human activity,” and “Genetic modification of plants improves the productivity of farming without posing health risks for consumers.” Importantly, each of the six items reiterated that the presented statement was made by scientists, but no information was given about the extent of scientific consensus. Indeed, not all statements in the survey are based on scientific consensus because most economists would contest the claim about tax revenue as it was phrased in the CRONOS-2 panel. Respondents answered each of the 15 questions on an 11-point scale ranging from 0 (No trust at all) to 10 (Complete trust).

This battery from the CRONOS-2 panel was linked to original items from the ESS Round 10. On the same aforementioned 11-point scale, ESS-respondents rated their trust in seven institutions that can be considered part of the system and the elite: the national parliament, the legal system, the police, politicians, political parties, the European Parliament, and the United Nations. The ESS also asked respondents to indicate their left–right identification on an 11-point scale ranging from 0 (Left) to 10 (Right). This scale can be considered a European equivalent of the liberal-conservative dimension that is typically used to measure ideology in the American context (Fuchs and Klingemann, 1990). Populist voting was measured with an item that asks respondents which party they voted for in the last national election. Based on their answer, respondents were assigned a score that indicates how populist the party they voted for is on a scale from 0 to 10. These scores were obtained from the “Populism and Political Parties Expert Survey” (POPPA). Drawing from the ‘ideational approach’ to populism, country-experts rated 250 parties from 28 countries on five ideological dimensions that Meijers and Zaslove (2021: 375) defined as follows: “Political sovereignty should reside with the ordinary people (1), the ordinary people are an indivisible or homogenous community (2) whose interests are united by a general will (3), the elite is portrayed as corrupt, (4) and the juxtaposition between the ordinary people and the elite is of Manichean proportions (5)”. The questionnaires can be found in Supplemental Appendix 1.

## 4. Analyses and results

**Figure 3.**
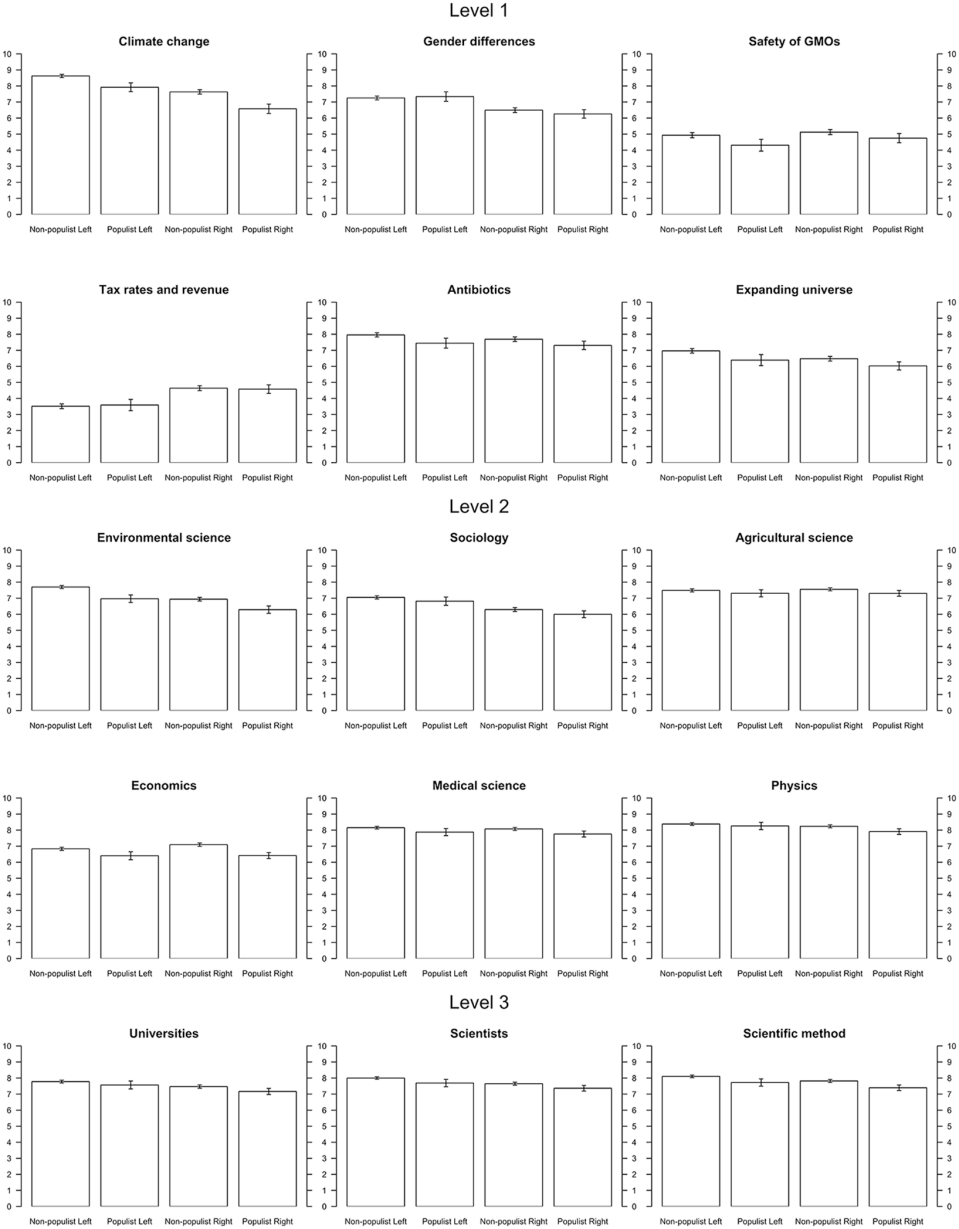
Descriptive results (weighted average of trust without control variables). Left: Left–right identification < 5; Right: Left–right identification > 5; Non-populist: POPPA-score < 5; Populist: POPPA-score > 5.

To provide some context for the main analysis, Table 1 displays the correlations between all variables, while Figure 3 depicts the mean scores on all science items—split out by political leaning. What stands out is that each item is, to some extent, connected to either populist voting or left–right identification, but that the overall level of trust in science is nonetheless high across the board. Unsurprisingly, the item about climate change clearly reveals the strongest level of science polarization. The items about tax revenue and the safety of GMOs are characterized by the lowest average levels of trust. The skepticism about the item on tax rates and tax revenue is unsurprising given that its phrasing in the CRONOS-2 panel does not align with the views of most economists. Respondents’ lack of trust in the safety of GM foods is, however, more remarkable given the scientific consensus on this issue (National Academies of Sciences, Engineering, and Medicine, 2016).

**Table 1.** Bivariate correlations.

	2	3	4	5	6	7	8	9	10	11	12	13	14	15	16
1. Populist vote	.09***	-.25***	-.15***	-.17***	-.11***	-.09***	-.13***	-.09***	-.09***	-.16***	-.08***	-.05***	-.00	-.06***	-.08***
2. Left–right identification		-.05**	-.10***	-.19***	-.19***	.01	.03	-.03*	-.04**	-.21***	-.15***	.03*	.17***	-.05**	-.11***
3. Trust in “The system and the elite” (scale score)			.35***	.35***	.29***	.28***	.29***	.30***	.25***	.26***	.13***	.19***	.04**	.14***	.16***
4. Trust in “Science as a whole” (scale score)				.59***	.57***	.57***	.54***	.65***	.65***	.41***	.27***	.26***	.03*	.34***	.34***
5. Trust in field: Environmental science					.56***	.61***	.50***	.53***	.49***	.50***	.29***	.21***	.03	.27***	.30***
6. Trust in field: Sociology						.42***	.50***	.40***	.41***	.34***	.30***	.12***	.04*	.21***	.22***
7. Trust in field: Agricultural science							.53***	.60***	.54***	.28***	.20***	.24***	.07***	.26***	.25***
8. Trust in field: Economics								.50***	.50***	.25***	.17***	.19***	.13***	.21***	.17***
9. Trust in field: Medical science									.59***	.34***	.23***	.27***	.05***	.28***	.24***
10. Trust in field: Physics										.30***	.16***	.17***	-.03*	.37***	.35***
11. Trust in claim: Climate change											.28***	.18***	-.01	.31***	.29***
12. Trust in claim: Gender differences												.11***	.10***	.14***	.17***
13. Trust in claim: Safety of GMOs													.22***	.18***	.28***
14. Trust in claim: Tax rates and revenue														.06***	.13***
15. Trust in claim: Antibiotics															.32***
16. Trust in claim: Expanding universe															

Controlled for country dummies but not for other variables.

* p < .05; ** p < .01; *** p < .001.

The hypotheses are discussed in reverse order, starting with hypothesis 5 about the model fit. The main analysis consists of two structural equation models—a hypothesized model and a fully parametrized model—that were estimated using “robust maximum likelihood” (MLR). The *hypothesized model* consists of all paths depicted in Figure 2, and the following parameters: the effect of country dummies and demographic control variables (i.e. gender, age, education, income, and religion) on all 22 trust items, residual correlations between the six research fields, residual correlations between the six scientific claims, and correlations between all independent variables (i.e. populist vote, left–right identification, country dummies, and demographic controls). The control variables ensure that the model accounts for country differences and basic demographic characteristics, while the residual correlations ensure that associations that do not contradict the logic of the model can be estimated freely. Three additional residual correlations were added because they revealed a value of over 500 in the model’s modification indices: between trust in the police and trust in the legal system, between politicians and political parties, and between the European Parliament and the United Nations. The resulting model yields a good fit (χ^2^(208) = 2375.205, p < .001, RMSEA = .044, CFI = .962). This indicates that the model in Figure 2 can accurately account for (most of) the correlations between the observed variables, which supports the hypothesis (H5) that the hypothesized four-level model would provide an accurate and parsimonious representation of how a variety of trust indicators relate to each other and to political leaning.

**Figure 4.**
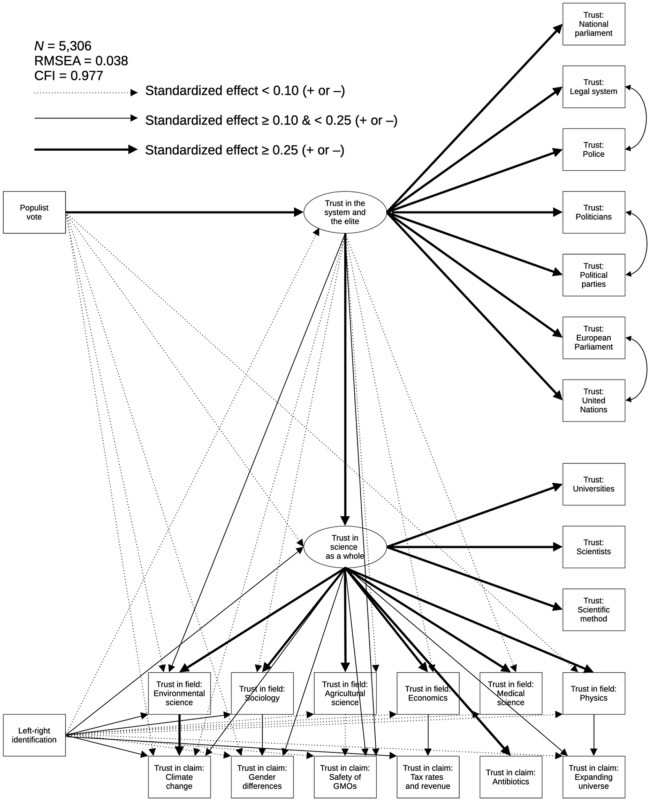
Estimated fully parameterized model. Estimates are displayed in Table 2. Model parameters not in the figure: the effect of country dummies on all 22 trust items, the effect of demographic control variables (i.e. gender, age, education, income, and religion) on all 22 trust items, residual correlations between the six research fields, residual correlations between the six scientific claims, and correlations between all independent variables (i.e. populist vote, left–right identification, all country dummies, and all demographic control variables).

The *fully parameterized model* includes all parameters of the hypothesized model, but every outcome of interest is now predicted by both populist voting and left–right identification as well as by trust on all more generalized levels. These additional parameters serve two purposes. First, they enable a more stringent test of the hypotheses by controlling each effect for trust on *all* more generalized levels rather than only the adjacent level. Second, this model can test if paths that were not hypothesized are indeed nonsignificant. The fully parameterized model—which again has a good fit (χ^2^(173) = 1508.672, p < .001, RMSEA = .038, CFI = .977)—is depicted in Figure 4 with an overview of parameter estimates in Table 2. These estimates provide additional support for hypothesis 5 because nearly all parameters that were not expected indeed turn out to be either nonsignificant or trivial in terms of effect size. The only added paths with considerable parameter estimates are the direct effects of trust in science as a whole on trust in specific claims. This suggests that when citizens determine their trust in a scientific statement, they use their trust in science as a whole as a heuristic in addition to their trust in the field that made the claim.

**Table 2.** Standardized estimates from the fully parameterized model in Figure 4.

	Hypothesized	b	SE	p-value
Polarization Level 1				
Left–right identification -> Climate change	Yes (H1a)	–0.12	0.01	< .001
Left–right identification -> Gender differences	Yes (H1a)	–0.09	0.01	< .001
Left–right identification -> Safety of GMOs	Yes (H1b)	0.05	0.01	< .001
Left–right identification -> Tax rates and revenue	Yes (H1b)	0.16	0.02	< .001
Left–right identification -> Antibiotics	No (H1c)	–0.01	0.01	.505
Left–right identification -> Expanding universe	No (H1c)	–0.06	0.01	< .001
Populist vote -> Climate change	No	–0.06	0.02	.001
Populist vote -> Gender differences	No	–0.04	0.02	.049
Populist vote -> Safety of GMOs	No	0.00	0.02	.956
Populist vote -> Tax rates and revenue	No	–0.01	0.02	.649
Populist vote -> Antibiotics	No	0.02	0.02	.290
Populist vote -> Expanding universe	No	–0.02	0.02	.322
Polarization Level 2				
Left–right identification -> Environmental science	Yes (H2a)	–0.10	0.01	< .001
Left–right identification -> Sociology	Yes (H2a)	–0.11	0.01	< .001
Left–right identification -> Agricultural science	Yes (H2b)	0.08	0.01	< .001
Left–right identification -> Economics	Yes (H2b)	0.09	0.01	< .001
Left–right identification -> Medical science	No (H2c)	0.04	0.01	.001
Left–right identification -> Physics	No (H2c)	0.02	0.01	.028
Populist vote -> Environmental science	No	–0.05	0.02	.006
Populist vote -> Sociology	No	0.00	0.02	.804
Populist vote -> Agricultural science	No	0.02	0.02	.202
Populist vote -> Economics	No	–0.03	0.02	.105
Populist vote -> Medical science	No	0.03	0.02	.121
Populist vote -> Physics	No	0.04	0.02	.032
Polarization Level 3				
Left–right identification -> Science as a whole	Yes (H3a)	–0.10	0.02	< .001
Populist vote -> Science as a whole	Yes (H3b)	–0.06	0.03	.017
Polarization Level 4				
Left–right identification -> The system and the elite	No	–0.05	0.02	.007
Populist vote -> The system and the elite	Yes (H4)	–0.30	0.02	< .001
Application of general contestation				
The system and the elite - > Science as a whole	Yes	0.33	0.02	< .001
The system and the elite - > Environmental science	No	0.12	0.01	< .001
The system and the elite - > Sociology	No	0.08	0.01	< .001
The system and the elite - > Agricultural science	No	0.07	0.01	< .001
The system and the elite - > Economics	No	0.09	0.01	< .001
The system and the elite - > Medical science	No	0.06	0.01	< .001
The system and the elite - > Physics	No	0.01	0.01	.714
The system and the elite - > Climate change	No	0.06	0.01	< .001
The system and the elite - > Gender differences	No	0.02	0.02	.307
The system and the elite - > Safety of GMOs	No	0.12	0.02	< .001
The system and the elite - > Tax revenue	No	0.03	0.02	.104
The system and the elite - > Antibiotics	No	–0.01	0.02	.610
The system and the elite - > Expanding universe	No	0.03	0.02	.060
Science as a whole - > Environmental science	Yes	0.52	0.02	< .001
Science as a whole - > Sociology	Yes	0.48	0.02	< .001
Science as a whole - > Agricultural science	Yes	0.52	0.02	< .001
Science as a whole - > Economics	Yes	0.47	0.02	< .001
Science as a whole - > Medical science	Yes	0.62	0.02	< .001
Science as a whole - > Physics	Yes	0.59	0.02	< .001
Science as a whole - > Climate change	No	0.18	0.02	< .001
Science as a whole - > Gender differences	No	0.16	0.02	< .001
Science as a whole - > Safety of GMOs	No	0.16	0.02	< .001
Science as a whole - > Tax revenue	No	–0.02	0.02	.210
Science as a whole - > Antibiotics	No	0.30	0.02	< .001
Science as a whole - > Expanding universe	No	0.21	0.02	< .001
Environmental science -> Climate change	Yes	0.30	0.02	< .001
Sociology -> Gender differences	Yes	0.17	0.02	< .001
Agricultural science -> Safety of GMOs	Yes	0.08	0.02	< .001
Economics -> Tax rates and revenue	Yes	0.14	0.02	< .001
Medical science -> Antibiotics	Yes	0.02	0.02	.440
Physics -> Expanding universe	Yes	0.14	0.02	< .001
Factor loadings				
The system and the elite -> National parliament	Yes	0.72	0.01	< .001
The system and the elite -> Legal system	Yes	0.57	0.01	< .001
The system and the elite -> Police	Yes	0.43	0.02	< .001
The system and the elite -> Politicians	Yes	0.73	0.01	< .001
The system and the elite -> Political parties	Yes	0.70	0.01	< .001
The system and the elite -> European Parliament	Yes	0.70	0.01	< .001
The system and the elite -> United Nations	Yes	0.59	0.01	< .001
Science as a whole -> Universities	Yes	0.60	0.01	< .001
Science as a whole -> Scientists	Yes	0.80	0.02	< .001
Science as a whole -> Scientific method	Yes	0.76	0.01	< .001
Residual correlations				
Legal system <-> Police	No	0.20	0.01	< .001
Politicians <-> Political parties	No	0.17	0.01	< .001
European Parliament <-> United Nations	No	0.19	0.01	< .001

The column “Hypothesized” indicates whether the estimated parameter was part of the hypothesized model in Figure 2.

The results in Figure 4 and Table 2 also confirm hypothesis 4 because respondents who cast their vote for a populist party indeed express considerably less trust (b = -.30) in the system and the elite than non-populist voters. Unexpectedly, people on the political right also trust elites and institutions slightly (-.05) less than left-wingers. Citizens’ trust in the system and the elite, in turn, reveals an effect of .33 on trust in science as a whole. This effect size appears particularly strong when considering that both constructs were measured at different time points. This finding underlines that science skepticism cannot be fully understood without considering a broader distrust in the elite and its institutions. After controlling for this effect, populist voters (–.06) and right-wingers (–.10) also express less trust in science as a whole, which confirms hypothesis 3.

Moving to the next level, this general trust in science shows strong effects (between .47 and .62) on respondents’ trust in each of the six research fields. After accounting for these effects, left–right identification still displays negative effects on trust in environmental science (-.10) and sociology (-.11), which confirms hypothesis 2a. Hypothesis 2b is, in turn, corroborated by the positive effects of left–right identification on trust in agricultural science (.08) and economics (.09). Interestingly, Table 1 points out that these effects do not exist at a bivariate level, so left-wingers are not more skeptical about production sciences in absolute terms, but they *do* trust these fields less than should be expected given their high general trust in science, while right-wingers trust these fields more than should be expected. Unexpectedly, right-wingers express more trust in medical science (.04) and physics (.02) than left-wingers, but the tiny magnitude of these effects is in line with the hypothesis (H2c) that both groups would trust nonpoliticized fields equally. As expected, populist voting barely displays direct effects on trust in research fields, with the exception of a small positive effect on trust in physics (.04) and a small negative effect for environmental science (–.05). Taken together, these patterns arguably provide some of the strongest evidence to date for McCright et al.’s (2013) thesis that conservatives and liberals are respectively prone to distrust impact science and production science.

At the level of specific claims, respondents’ trust in the six scientific statements is predicted by their trust in the field that made the claim, as well as by their general trust in science. After controlling for these effects, right-wingers still indicate less trust than left-wingers in the claims about climate change (–.12) and gender differences (–.09), which confirms hypothesis 1a. The results also corroborate hypothesis 1b by—unlike most previous studies—revealing that left-wingers are indeed more skeptical than right-wingers about liberal-dissonant claims such as the safety of GM foods (.05) and the relationship between tax rates and tax revenue (.16). Interestingly, Table 1 shows that these associations also exist at a bivariate level, albeit with a smaller effect size for the GMO claim. As was the case for research fields, this again indicates that science rejection looks more symmetric between liberals and conservatives after accounting for more generalized levels of trust. Hypothesis 1c about neutral claims is largely, but not entirely, supported—right-wingers express slightly less trust than left-wingers in the claim about the expanse of the universe (–.06) but, as hypothesized, both groups trust the claim about antibiotics equally. Also as expected, populist voting did not display direct effects on respondents’ trust in specific claims, with the exception of small negative effects for climate change (–.06) and gender differences (–.04). This means that the science polarization between populist and non-populist voters over research fields and specific claims (see Table 1 and Figure 3) is—with a few exceptions—indeed accounted for by populists’ distrust on more generalized levels, which again corroborates the proposed theoretical framework (see Figures 1 and 2). The small direct effects of populist voting on trust in claims about climate change and gender differences could, tentatively, be explained by the emphasis that many right-wing populist parties place on these issues in their anti-scientific discourse.

**Figure 5.**
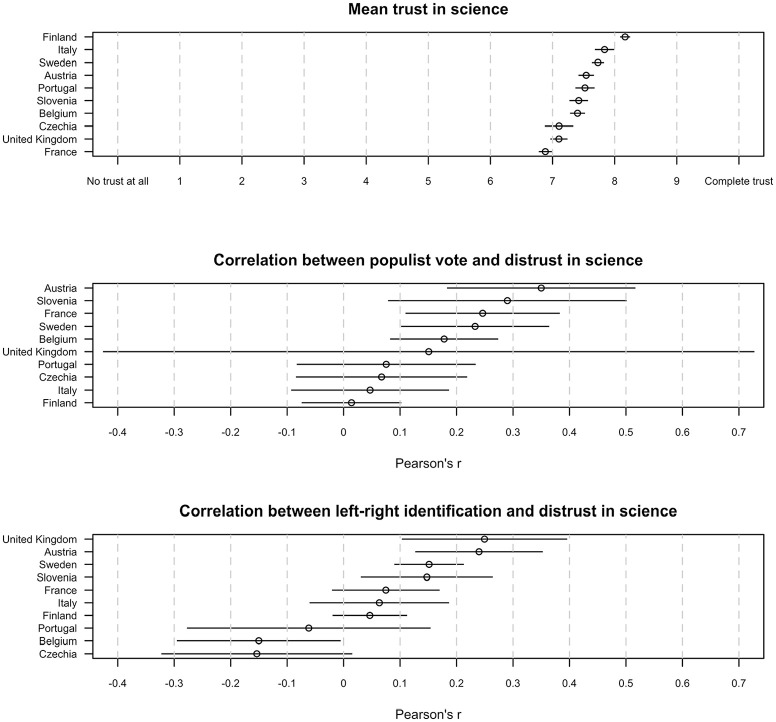
Exploratory country comparison. Based on scale scores on trust in science (i.e. mean scores on trust in universities, scientists, and the scientific method), scores are reverse-coded to “distrust in science” for the correlations. The results in this figure are weighted but not controlled for third variables.

The structural equation models could only be estimated across all 10 countries simultaneously, because the sample size in each separate country is too small in relation to the number of parameters. Although this makes a full country-comparison impossible, Figure 5 depicts some exploratory analyses on country differences at the level of trust in science as a whole. The average trust in science seems highest in Finland, Italy, and Sweden and lowest in Czechia, the United Kingdom, and France. Country-differences in the science polarization between populist and non-populist voters are mostly nonsignificant, but Figure 5 clearly shows that the sample includes too few British populist voters to provide a meaningful estimate for this country. The effect of the left–right dimension, however, differs more between countries, which is unsurprising given the well-known country-differences in the strength and meaning of this dimension (Otjes and Rekker, 2021). In Belgium, right-wingers notably express slightly more trust in science than left-wingers. This cross-country variation echoes the findings of a 67-country survey, which also pointed out that the effect of left–right identification on trust in science varies between countries in both strength and direction (Cologna et al., 2024). The general picture that emerges from Figure 5 is, however, that the same general patterns exist across the 10 countries in this study and that most country-differences in science polarization are nonsignificant. The analyses finally include two robustness checks. Supplemental Appendix 2 demonstrates that none of the main findings depend on the inclusion or exclusion of demographic control variables. The analysis in Supplemental Appendix 3, in turn, establishes that the same patterns and similar effect sizes emerge when modeling trust in “the system and the elite” and “science as a whole” as simple scale scores, which means that the results do not depend on the particular causal assumptions of latent variable modeling (Edwards and Bagozzi, 2000).

## 5. Discussion

Citizens’ trust in science increasingly depends on their political leaning. Rekker (2021) argued that this science polarization can originate from distrust on four distinct levels of generalization: specific claims, research fields, science as a whole, and the system and elite. By examining the politicization of trust on all four levels simultaneously across 10 European countries, this study provides the first complete empirical test of this four-level model. The good fit of this model on the data establishes that—even though other models may also fit the data—the hypothesized model provides a sufficiently accurate and parsimonious representation of how a variety of trust indicators relate to each other and to citizens’ political leaning. The strong associations between the four levels furthermore support the idea that people use more generalized levels of trust as a heuristic to form more specific attitudes (i.e. application of general contestation; Rekker, 2021). Citizens’ trust in human-made climate change, for instance, seems to depend on their trust in environmental science (.30), which in turn depends on their trust in science as a whole (.52), which in turn depends on their trust in the system and elite (.33). In addition, people also seem to use their general trust in science as a direct heuristic for their trust in anthropogenic global warming (.18). This underlines that studies on science skepticism should carefully consider all four levels of trust and their interplay when determining what outcome variable and control variables should be examined for a particular research question.

With that said, the limitations of the current examination should also be considered, particularly with regard to the direction of causality. Importantly, the cross-sectional design of this study cannot disentangle the effect of one variable on another from the effect in the opposite direction. Panel studies, for instance, indicate that political discontent predicts populist voting as much as vice versa (Rekker, 2023; Rooduijn et al., 2016). The effect in the current study of populist voting on trust in the system and the elite should, therefore, be interpreted as a bidirectional process. Longitudinal research, however, also indicates that populist voting predicts science skepticism over time but not the other way around (Rekker, 2023), so the effect of populist voting on the other three levels of generalization could be more unidirectional. Another process that could be bidirectional involves the way in which trust travels between the four levels of generalization. Rekker (2021) theorized that citizens who reject science on specific issues may resultingly become distrustful of science as a whole (i.e. *generalization of specific contestation*), as well as vice versa (i.e. *application of generalized contestation*, see Figure 1). Constrained by its cross-sectional design, this study could not disentangle both processes. All associations between the four levels are, therefore, modeled in the direction of “application of generalized contestation” because the empirical evidence for this mechanism seems slightly stronger than for the effect of “generalization of specific contestation” (Brossard and Nisbet, 2007; Hmielowski et al., 2014). Moreover, a panel study that compared both effects found that generalized distrust predicts specific distrust much more than vice versa (Rekker, 2023). In other words, the available evidence carefully suggests that the associations between the four levels in this study run predominantly, but not exclusively, in the modeled direction.

The main theoretical contribution of this study lies in its ability to—assuming a direction of causality in cross-sectional data—trace back science polarization to the level of contestation from which it originates. The results demonstrate that the science polarization between populist and non-populist voters has fundamentally different roots than the effect of left–right ideology. For populist voters, science rejection seems mostly (but not exclusively) a byproduct of their general distrust in the elite and its institutions. In contrast, distrust among left-wingers and right-wingers arises because specific claims and research fields challenge their ideological worldview. This distinction may inform strategies to counteract science polarization. The gap between left-wingers and right-wingers may be narrowed by science communication strategies that make scientific claims more congruent with a political ideology (Kahan et al., 2015). The notion of anthropogenic global warming may, for instance, become more acceptable for right-wingers when the benefits of the energy transition for economic growth are communicated. In a similar vein, science communication could inform left-wingers about the potential of GMOs to improve food security in developing nations. In contrast, reducing the science polarization between populist and non-populist voters may require a much broader agenda to restore institutional trust by bridging the gap between elites and ordinary citizens.

Finally, this examination established that a four-level model of polarization can expose ideological symmetries in science rejection that have previously remained largely undetected in observational studies. So far, the literature has been characterized by a paradox that, on the one hand, experimental studies have generally pointed out that liberals and conservatives are equally biased against counterattitudinal information (Ditto et al., 2019) while, on the other hand, observational research has quite consistently demonstrated that liberals have more trust in scientific claims (e.g. Hunt and Wald, 2020)—as well as more accurate beliefs (e.g. Rutjens et al., 2018)—even on issues that seemingly contradict their ideology. This study revealed that both findings may be reconciled by distinguishing the *direct effect* of political ideology on domain-specific science skepticism from an *indirect effect* that runs via more generalized levels of distrust. The indirect effect is characterized by *ideological asymmetry*, because liberals have more trust in science as a whole which, in turn, strengthens their trust in all fields and claims, regardless of content. Contrarily, the direct effect displays *ideological symmetry* because both liberals and conservatives adjust this baseline trust in a downward direction when a field or claim challenges their ideology.

## Supplemental Material

sj-pdf-1-pus-10.1177_09636625241306352 – Supplemental material for A four-level model of political polarization over science: Evidence from 10 European countries Supplemental material, sj-pdf-1-pus-10.1177_09636625241306352 for A four-level model of political polarization over science: Evidence from 10 European countries by Roderik Rekker in Public Understanding of Science
