# Supplementary material for: A four-level model of political polarization over science: Evidence from 10 European countries
Source: Public Underst Sci. 2025 Jan 18;34(4):424–45. doi: 10.1177/09636625241306352 (PMC12038069; doi:10.1177/09636625241306352)
Supplement: sj-pdf-1-pus-10.1177_09636625241306352 – Supplemental material for A four-level model of political polarization over science: Evidence from 10 European countries [file sj-pdf-1-pus-10.1177_09636625241306352.pdf]

A four-level model of political polarization over science:
Evidence from 10 European countries

Roderik Rekker

Radboud University, the Netherlands
University of Gothenburg, Sweden
Leibniz University Hannover, Germany

Supplemental Material Contents

Supplemental Material 1: Questionnaires.
Supplemental Material 2: Standardized estimates from the fully parameterized model without control variables.
Supplemental Material 3: Standardized estimates from the fully parameterized model with the latent variables of 'The system and the elite' and 'Science as a whole' replaced by scale scores of their indicators.

Supplemental Material 1A:
Relevant pages from the ESS Round 10 questionnaire
(United Kingdom)

CARD 9 Using this card, please tell me on a score of 0-10 how much you personally trust each of the institutions I read out. 0 means you do not trust an institution at all, and 10 means you have complete trust. Firstly… **READ OUT**…

[Display before items B7-B12: STILL CARD 9 (IF NECESSARY: How much do you trust…) **READ OUT…]**

		No trust at all										Complete trust	(Refusal)	(Don't know)
**B6**	…[Britain / the UK[9]]'s parliament?	00	01	02	03	04	05	06	07	08	09	10	77	88
**B7**	…the legal system?	00	01	02	03	04	05	06	07	08	09	10	77	88
**B8**	…the police?	00	01	02	03	04	05	06	07	08	09	10	77	88
**B9**	…politicians?	00	01	02	03	04	05	06	07	08	09	10	77	88
**B10**	…political parties?	00	01	02	03	04	05	06	07	08	09	10	77	88
**B11**	…the European Parliament?	00	01	02	03	04	05	06	07	08	09	10	77	88
**B12**	…the United Nations?	00	01	02	03	04	05	06	07	08	09	10	77	88
**(Trstsci) B12a**	…scientists[10]	00	01	02	03	04	05	06	07	08	09	10	77	88

B13 Some people don't vote nowadays for one reason or another. Did you vote in the last [British [11] /general] election in [December 2019]?

Yes	1	**ASK B14**
No	2	
Not eligible to vote	3	**GO TO B15**
(Refusal)	7	
(Don't know)	8	

[9] In England, Scotland & Wales the questionnaire referred to Britain, in Northern Ireland it referred to the UK

[10] This item forms part of the COVID-19 module. It should therefore only be included for countries including the COVID-19 module.

[11] In England, Scotland & Wales the questionnaire referred to the British general election, in Northern Ireland it referred to the general election

ASK IF YES AT B13 (IF B13 = 1)

B14 Which party did you vote for in that election?
[England, Scotland & Wales codes]

Conservative	01
Labour	02
Liberal Democrat	03
Scottish National Party	04
Plaid Cymru	05
Green Party	06
UK Independence Party	07
Brexit Party	08
Other (WRITE IN) ______________________________	09
(Refusal)	77
(Don't know)	88

ASK IF YES AT B13 (code 1)
B14 Which party did you vote for in that election?
[Northern Ireland codes]

Ulster Unionist Party	01
Democratic Unionist Party	02
Sinn Fein	03
Social Democratic and Labour Party	04
Alliance Party of Northern Ireland	05
Traditional Unionist Voice	06
Green Party in Northern Ireland	07
Independent(s)	08
People Before Profit Alliance	09
Progressive Unionist Party	10
UK Independence Party	11
Aontú	12
Northern Ireland Conservatives	13
Other (WRITE IN) ______________________________	~~14~~
(Refused)	77
(Don't know)	88

ASK ALL
There are different ways of trying to improve things in [Britain / the UK[12]] or help prevent things from going wrong[13]. During the last 12 months, have you done any of the following?
Have you… **READ OUT**…

[Display before items B16-B18: (**IF NECESSARY:** During the last 12 months, have you…) **READ OUT**…**]**

[12] In England, Scotland & Wales the questionnaire referred to Britain, in Northern Ireland it referred to the UK

[13] 'Help prevent things from going wrong' in the sense of help prevent serious problems arising.

ASK ALL
implvdm
B25a[23] **CARD 10** How important is it for you to live in a country that is governed democratically? Choose your answer from this card where 0 is not at all important and 10 is extremely[24] important.

Not at all important										Extremely important	(Refusal)	(Don't know)
00	01	02	03	04	05	06	07	08	09	10	77	88

B26 **CARD 11** In politics people sometimes talk of "left" and "right". Using this card, where would you place yourself on this scale, where 0 means the left and 10 means the right?

Left										Right	(Refusal)	(Don't know)
00	01	02	03	04	05	06	07	08	09	10	77	88

B27 **CARD 12** All things considered, how satisfied are you with your life as a whole nowadays? Please answer using this card, where 0 means extremely[25] dissatisfied and 10 means extremely satisfied.

Extremely dissatisfied										Extremely satisfied	(Refusal)	(Don't know)
00	01	02	03	04	05	06	07	08	09	10	77	88

B28 **STILL CARD 12** On the whole how satisfied are you with the present state of the economy in [Britain / the UK[26]]? Still use this card.

Extremely dissatisfied										Extremely satisfied	(Refusal)	(Don't know)

[23] **REPEAT QUESTION** from the ESS6 module on Democracy. Please refer to item B18d in ESS6 for translation.
[24] 'Extremely' in the sense of 'an end point on the scale where nothing can go beyond it. This might also be represented by 'completely', 'fully', 'absolutely', 'totally', etc.
[25] Please note that for CORE items using 'extremely' or 'completely', the same translation should be used as in previous rounds. This applies throughout sections B, C, F and I. If translators are unsure, contact ess_translate@gesis.org.
[26] In England, Scotland & Wales the questionnaire referred to British government, in Northern Ireland it referred to the government of the UK

Supplemental Material 1B:
Relevant pages from the CRONOS-2 questionnaire
(United Kingdom)

Question Block	Question Number
Introduction	T1

Welcome back to The Opinion Study for the UK and thank you in advance for answering the survey. This time we will ask about trust in universities and scientists, your experiences and feelings when you meet with other people, access to various services during the COVID-19 pandemic in the UK, and things that might influence people's earnings.

- If you don't want to answer a question just leave it blank and click the button at the bottom right of the screen to move on
- Your data will be handled in the strictest confidence, in accordance with the General Data Protection Regulation and only used for research

Please, click the button below to start the survey

→

Powered by Qualtrics

Question Block	Question Number
In science we trust	w5q1

Let's start with some questions about how much trust you have in different institutions or activities.

In general, how much do you personally trust universities?
Please choose an answer between 0 and 10, where 0 is "No trust at all" and 10 is "Complete trust".

- ○ 0 - No trust at all
- ○ 1
- ○ 2
- ○ 3
- ○ 4
- ○ 5
- ○ 6
- ○ 7
- ○ 8
- ○ 9
- ○ 10 - Complete trust

→

Powered by Qualtrics

Question Block	Question Number
In science we trust	w5q2

In general, how much do you personally trust scientists?

- 0 - No trust at all
- 1
- 2
- 3
- 4
- 5
- 6
- 7
- 8
- 9
- 10 - Complete trust

← →

Powered by Qualtrics

Question Block	Question Number
In science we trust	w5q3

In general, how much do you personally trust scientific methods?

- 0 - No trust at all
- 1
- 2
- 3
- 4
- 5
- 6
- 7
- 8
- 9
- 10 - Complete trust

← →

Powered by Qualtrics

Question Block	Question Number
In science we trust	w5q4

The next questions are about how much trust you have in specific scientific disciplines.

How much trust do you have in this scientific discipline: Sociology

Please choose an answer between 0 and 10, where 0 is "No trust at all" and 10 is "Complete trust". Remember that if you do not know how to answer a question, you can leave it blank and move to the next one.

- 0 - No trust at all
- 1
- 2
- 3
- 4
- 5
- 6
- 7
- 8
- 9
- 10 - Complete trust

→

Powered by Qualtrics

Question Block	Question Number
In science we trust	w5q5

How much trust do you have in this scientific discipline: Economics

- ◯ 0 - No trust at all
- ◯ 1
- ◯ 2
- ◯ 3
- ◯ 4
- ◯ 5
- ◯ 6
- ◯ 7
- ◯ 8
- ◯ 9
- ◯ 10 - Complete trust

← →

Powered by Qualtrics

Question Block	Question Number
In science we trust	w5q6

How much trust do you have in this scientific discipline: Physics

- 0 - No trust at all
- 1
- 2
- 3
- 4
- 5
- 6
- 7
- 8
- 9
- 10 - Complete trust

← →

Powered by Qualtrics

Question Block	Question Number
In science we trust	w5q7

How much trust do you have in this scientific discipline: Medical science

- 0 - No trust at all
- 1
- 2
- 3
- 4
- 5
- 6
- 7
- 8
- 9
- 10 - Complete trust

← →

Powered by Qualtrics

Question Block	Question Number
In science we trust	w5q8

How much trust do you have in this scientific discipline: Agricultural science

- 0 - No trust at all
- 1
- 2
- 3
- 4
- 5
- 6
- 7
- 8
- 9
- 10 - Complete trust

← →

Powered by Qualtrics

Question Block	Question Number
In science we trust	w5q9

How much trust do you have in this scientific discipline: Environmental science

- ◯ 0 - No trust at all
- ◯ 1
- ◯ 2
- ◯ 3
- ◯ 4
- ◯ 5
- ◯ 6
- ◯ 7
- ◯ 8
- ◯ 9
- ◯ 10 - Complete trust

← →

Powered by Qualtrics

Question Block	Question Number
In science we trust	w5q10

The next questions are about statements which scientists from different disciplines have made.

<u>How much trust do you have in this statement made by scientists:</u>
Differences in behaviour between men and women are not fixed at birth but are mainly caused by upbringing of parents and society.

- 0 - No trust at all
- 1
- 2
- 3
- 4
- 5
- 6
- 7
- 8
- 9
- 10 - Complete trust

→

Powered by Qualtrics

Question Block	Question Number
In science we trust	w5q11

How much trust do you have in this statement made by scientists:
Increasing taxes on the rich will reduce government revenue because rich people will work less.

- 0 - No trust at all
- 1
- 2
- 3
- 4
- 5
- 6
- 7
- 8
- 9
- 10 - Complete trust

← →

Powered by Qualtrics

Question Block	Question Number
In science we trust	w5q12

How much trust do you have in this statement made by scientists:

The universe expands at an increasing rate.

- 0 - No trust at all
- 1
- 2
- 3
- 4
- 5
- 6
- 7
- 8
- 9
- 10 - Complete trust

← →

Powered by Qualtrics

Question Block	Question Number
In science we trust	w5q13

How much trust do you have in this statement made by scientists:
Antibiotics do not work against viruses because they only kill bacteria.

- 0 - No trust at all
- 1
- 2
- 3
- 4
- 5
- 6
- 7
- 8
- 9
- 10 - Complete trust

← →

Powered by Qualtrics

Question Block	Question Number
In science we trust	w5q14

How much trust do you have in this statement made by scientists:

The Earth's climate is changing as a result of greenhouse gas emissions caused by human activity.

- 0 - No trust at all
- 1
- 2
- 3
- 4
- 5
- 6
- 7
- 8
- 9
- 10 - Complete trust

← →

Powered by Qualtrics

Question Block	Question Number
In science we trust	w5q15

How much trust do you have in this statement made by scientists:
Genetic modification of plants improves the productivity of farming without posing health risks for consumers.

- 0 - No trust at all
- 1
- 2
- 3
- 4
- 5
- 6
- 7
- 8
- 9
- 10 - Complete trust

← →

Powered by Qualtrics

Supplemental Material 2. *Standardized estimates from the fully parameterized model without control variables.*

	Hypothesized	b	SE	p-value
Polarization Level 1				
Left-right identification -> Climate change	Yes (H1a)	–0.13	0.01	<.001
Left-right identification -> Gender differences	Yes (H1a)	–0.10	0.01	<.001
Left-right identification -> Safety of GMOs	Yes (H1b)	0.05	0.01	<.001
Left-right identification -> Tax rates and revenue	Yes (H1b)	0.16	0.02	<.001
Left-right identification -> Antibiotics	No (H1c)	–0.01	0.01	.633
Left-right identification -> Expanding universe	No (H1c)	–0.07	0.01	<.001
Populist vote -> Climate change	No	–0.07	0.02	<.001
Populist vote -> Gender differences	No	–0.03	0.02	.094
Populist vote -> Safety of GMOs	No	0.01	0.02	.568
Populist vote -> Tax rates and revenue	No	–0.00	0.02	.906
Populist vote -> Antibiotics	No	0.00	0.02	.872
Populist vote -> Expanding universe	No	–0.02	0.02	.224
Polarization Level 2				
Left-right identification -> Environmental science	Yes (H2a)	–0.11	0.01	<.001
Left-right identification -> Sociology	Yes (H2a)	–0.13	0.01	<.001
Left-right identification -> Agricultural science	Yes (H2b)	0.08	0.01	<.001
Left-right identification -> Economics	Yes (H2b)	0.09	0.01	<.001
Left-right identification -> Medical science	No (H2c)	0.05	0.01	<.001
Left-right identification -> Physics	No (H2c)	0.03	0.01	.008
Populist vote -> Environmental science	No	–0.04	0.02	.008
Populist vote -> Sociology	No	0.01	0.02	.605
Populist vote -> Agricultural science	No	0.03	0.02	.086
Populist vote -> Economics	No	–0.02	0.02	.279
Populist vote -> Medical science	No	0.02	0.02	.148
Populist vote -> Physics	No	0.03	0.02	.067
Polarization Level 3				
Left-right identification -> Science as a whole	Yes (H3a)	–0.09	0.02	<.001
Populist vote -> Science as a whole	Yes (H3b)	–0.05	0.02	.061
Polarization Level 4				
Left-right identification -> The system and the elite	No	–0.03	0.02	.042
Populist vote -> The system and the elite	Yes (H4)	–0.30	0.02	<.001
Application of general contestation				
The system and the elite - > Science as a whole	Yes	0.38	0.02	<.001
The system and the elite - > Environmental science	No	0.13	0.01	<.001
The system and the elite - > Sociology	No	0.09	0.01	<.001
The system and the elite - > Agricultural science	No	0.08	0.01	<.001
The system and the elite - > Economics	No	0.10	0.01	<.001
The system and the elite - > Medical science	No	0.06	0.01	<.001
The system and the elite - > Physics	No	0.02	0.01	.150
The system and the elite - > Climate change	No	0.06	0.01	<.001
The system and the elite - > Gender differences	No	0.01	0.02	.613
The system and the elite - > Safety of GMOs	No	0.12	0.02	<.001
The system and the elite - > Tax revenue	No	0.03	0.02	.146
The system and the elite - > Antibiotics	No	0.01	0.02	.707
The system and the elite - > Expanding universe	No	0.04	0.02	.032
Science as a whole - > Environmental science	Yes	0.52	0.02	<.001
Science as a whole - > Sociology	Yes	0.48	0.02	<.001
Science as a whole - > Agricultural science	Yes	0.54	0.02	<.001
Science as a whole - > Economics	Yes	0.48	0.02	<.001
Science as a whole - > Medical science	Yes	0.63	0.02	<.001
Science as a whole - > Physics	Yes	0.64	0.02	<.001
Science as a whole - > Climate change	No	0.18	0.02	<.001
Science as a whole - > Gender differences	No	0.15	0.02	<.001
Science as a whole - > Safety of GMOs	No	0.16	0.02	<.001
Science as a whole - > Tax revenue	No	–0.05	0.02	.005
Science as a whole - > Antibiotics	No	0.34	0.02	<.001
Science as a whole - > Expanding universe	No	0.20	0.02	<.001
Environmental science -> Climate change	Yes	0.30	0.02	<.001
Sociology -> Gender differences	Yes	0.18	0.02	<.001
Agricultural science -> Safety of GMOs	Yes	0.08	0.02	<.001
Economics -> Tax rates and revenue	Yes	0.16	0.02	<.001
Medical science -> Antibiotics	Yes	0.01	0.02	.536
Physics -> Expanding universe	Yes	0.18	0.02	<.001
Factor loadings				
The system and the elite -> National parliament	Yes	0.75	0.01	<.001
The system and the elite -> Legal system	Yes	0.61	0.01	<.001
The system and the elite -> Police	Yes	0.44	0.02	<.001
The system and the elite -> Politicians	Yes	0.74	0.01	<.001
The system and the elite -> Political parties	Yes	0.71	0.01	<.001
The system and the elite -> European Parliament	Yes	0.72	0.01	<.001
The system and the elite -> United Nations	Yes	0.61	0.01	<.001
Science as a whole -> Universities	Yes	0.62	0.01	<.001
Science as a whole -> Scientists	Yes	0.80	0.02	<.001
Science as a whole -> Scientific method	Yes	0.79	0.01	<.001
Residual correlations				
Legal system <-> Police	No	0.20	0.01	<.001
Politicians <-> Political parties	No	0.18	0.01	<.001
European Parliament <-> United Nations	No	0.20	0.01	<.001

Note. This alternative model is controlled for country dummies, but not demographic control variables. The column 'Hypothesized' indicates whether the estimated parameter was part of the hypothesized model in Figure 2.

Supplemental Material 3. *Standardized estimates from the fully parameterized model with the latent variables of 'The system and the elite' and 'Science as a whole' replaced by scale scores of their indicators.*

	Hypothesized	b	SE	p-value
Polarization Level 1				
Left-right identification -> Climate change	Yes (H1a)	–0.12	0.01	<.001
Left-right identification -> Gender differences	Yes (H1a)	–0.09	0.01	<.001
Left-right identification -> Safety of GMOs	Yes (H1b)	0.05	0.01	.001
Left-right identification -> Tax rates and revenue	Yes (H1b)	0.16	0.02	<.001
Left-right identification -> Antibiotics	No (H1c)	–0.02	0.01	.219
Left-right identification -> Expanding universe	No (H1c)	–0.07	0.01	<.001
Populist vote -> Climate change	No	–0.06	0.02	.001
Populist vote -> Gender differences	No	–0.04	0.02	.030
Populist vote -> Safety of GMOs	No	–0.01	0.02	.681
Populist vote -> Tax rates and revenue	No	–0.01	0.02	.635
Populist vote -> Antibiotics	No	0.01	0.02	.498
Populist vote -> Expanding universe	No	–0.02	0.02	.221
Polarization Level 2				
Left-right identification -> Environmental science	Yes (H2a)	–0.11	0.01	<.001
Left-right identification -> Sociology	Yes (H2a)	–0.12	0.01	<.001
Left-right identification -> Agricultural science	Yes (H2b)	0.07	0.01	<.001
Left-right identification -> Economics	Yes (H2b)	0.09	0.01	<.001
Left-right identification -> Medical science	No (H2c)	0.03	0.01	.019
Left-right identification -> Physics	No (H2c)	0.01	0.01	.210
Populist vote -> Environmental science	No	–0.06	0.02	.001
Populist vote -> Sociology	No	–0.00	0.02	.872
Populist vote -> Agricultural science	No	0.01	0.02	.478
Populist vote -> Economics	No	–0.04	0.02	.029
Populist vote -> Medical science	No	0.02	0.02	.319
Populist vote -> Physics	No	0.03	0.02	.119
Polarization Level 3				
Left-right identification -> Science as a whole	Yes (H3a)	–0.08	0.01	<.001
Populist vote -> Science as a whole	Yes (H3b)	–0.05	0.02	.012
Polarization Level 4				
Left-right identification -> The system and the elite	No	–0.03	0.01	.020
Populist vote -> The system and the elite	Yes (H4)	–0.24	0.02	<.001
Application of general contestation				
The system and the elite - > Science as a whole	Yes	0.32	0.02	<.001
The system and the elite - > Environmental science	No	0.16	0.01	<.001
The system and the elite - > Sociology	No	0.11	0.02	<.001
The system and the elite - > Agricultural science	No	0.09	0.02	<.001
The system and the elite - > Economics	No	0.11	0.02	<.001
The system and the elite - > Medical science	No	0.10	0.01	<.001
The system and the elite - > Physics	No	0.03	0.01	.063
The system and the elite - > Climate change	No	0.08	0.02	<.001
The system and the elite - > Gender differences	No	0.02	0.02	.262
The system and the elite - > Safety of GMOs	No	0.12	0.02	<.001
The system and the elite - > Tax revenue	No	0.03	0.02	.138
The system and the elite - > Antibiotics	No	–0.00	0.02	.950
The system and the elite - > Expanding universe	No	0.04	0.02	.029
Science as a whole - > Environmental science	Yes	0.54	0.01	<.001
Science as a whole - > Sociology	Yes	0.53	0.01	<.001
Science as a whole - > Agricultural science	Yes	0.55	0.02	<.001
Science as a whole - > Economics	Yes	0.61	0.02	<.001
Science as a whole - > Medical science	Yes	0.64	0.01	<.001
Science as a whole - > Physics	Yes	0.61	0.02	<.001
Science as a whole - > Climate change	No	0.18	0.02	<.001
Science as a whole - > Gender differences	No	0.17	0.02	<.001
Science as a whole - > Safety of GMOs	No	0.16	0.02	<.001
Science as a whole - > Tax revenue	No	–0.01	0.02	.710
Science as a whole - > Antibiotics	No	0.26	0.02	<.001
Science as a whole - > Expanding universe	No	0.21	0.02	<.001
Environmental science -> Climate change	Yes	0.31	0.02	<.001
Sociology -> Gender differences	Yes	0.18	0.02	<.001
Agricultural science -> Safety of GMOs	Yes	0.10	0.02	<.001
Economics -> Tax rates and revenue	Yes	0.13	0.02	<.001
Medical science -> Antibiotics	Yes	0.08	0.02	<.001
Physics -> Expanding universe	Yes	0.16	0.02	<.001

Note. Scale scores on 'The system and the elite' reflect respondents' mean score on trust in the national parliament, the legal system, the police, politicians, political parties, the European Parliament, and the United Nations. Scale scores on 'Science as a whole' reflect respondents' mean score on trust in universities, scientists, and the scientific method. The column 'Hypothesized' indicates whether the estimated parameter was part of the hypothesized model in Figure 2.
